# Increased Short-Term Fluctuation in Optic Nerve Head Blood Flow in a Case of Normal-Tension Glaucoma by the Use of Laser Speckle Flowgraphy

**DOI:** 10.3390/vision1010005

**Published:** 2016-09-08

**Authors:** Tetsuya Sugiyama, Hajime Nakamura

**Affiliations:** Nakano Eye Clinic of Kyoto Medical Co-Operative, Kyoto 604-8404, Japan

**Keywords:** normal-tension glaucoma, optic nerve head blood flow, short-term fluctuation, kallidinogenase

## Abstract

An 80-year-old woman with normal-tension glaucoma was transferred to our clinic 9 years ago. She exhibited progressive visual field defect despite intraocular pressure in both eyes remaining stable in the low teens after treatment with prostaglandin-derivative eye drops. Increased short-term fluctuation in optic nerve head (ONH) blood flow was detected using laser speckle flowgraphy. After the patient was administered kallidinogenase tablets, the fluctuation was reduced and her visual field defect was ameliorated. However, the fluctuation increased and the visual field defect deteriorated after the patient discontinued the medication. The increased short-term fluctuation in ONH blood flow seemed to be associated with the development of glaucomatous visual field defect in this case.

## 1. Introduction

Global surveys indicate that glaucoma is the second leading cause of visual impairment [1]. Most aspects of the pathogenesis of glaucoma, particularly normal-tension glaucoma (NTG), remain unclear, and the only evidence-based therapy for glaucoma is reducing intraocular pressure (IOP) [2,3,4]. Nevertheless, some cases of NTG progress in spite of sufficiently reduced IOP, suggesting that factors independent of IOP may be involved in its pathogenesis. Epidemiological studies have suggested that lower ocular perfusion pressure is associated with an increased prevalence or progression of glaucoma [5,6], and local and systemic vascular factors have been confirmed to play a role in the pathophysiology of glaucoma [7]. In addition, several reports have suggested a larger diurnal fluctuation in the parameters of ocular blood flow in patients with glaucoma, including NTG [8,9,10]. Evidence has also suggested that endothelin-1 and nitric oxide play roles in the vascular dysregulation, which is associated with the pathogenesis of glaucomatous optic neuropathy, particularly in NTG [11,12,13,14,15].

In the current report, we present a case of advanced-stage NTG with increased short-term fluctuation of optic nerve head (ONH) blood flow detected using laser speckle flowgraphy (LSFG).

## 2. Case Presentation

An 80-year-old woman had complained of bilateral visual field defect and had been diagnosed with NTG by an ophthalmologist in 2004. She had been receiving treatment with eye drops (unoprostone isopropyl) when she was transferred to our clinic 3 years after the first diagnosis. Her personal medical history included multiple cerebral infarctions and left mild atrial hypertrophy (Her systemic blood pressure was within normal range: 110~130/60~80). She underwent surgeries for cataracts in both eyes at our clinic in in the next year. She exhibited progressive visual field defect despite the IOP in both eyes remaining stable in the low teens after treatment with topical application of tafluprost.

**Figure 1:**
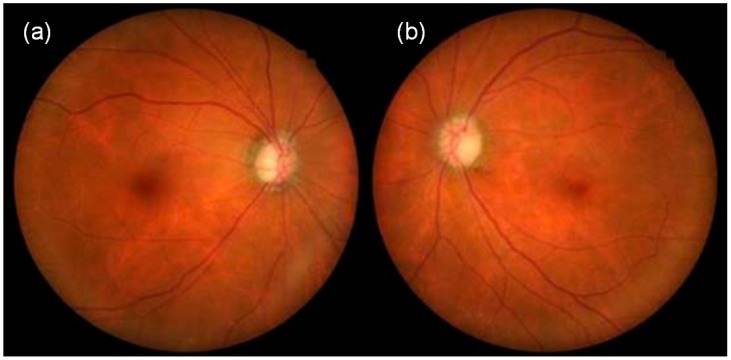
Fundus photographs at her visit to our clinic on 21 October 2013 (**a**: right eye, **b**: left eye).

**Figure 2:**
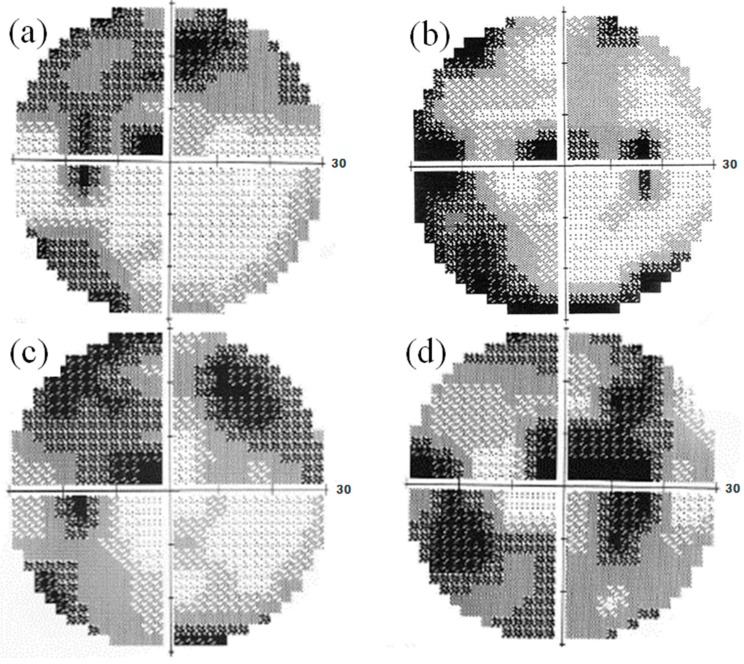
Visual field findings obtained using the Humphrey field analyzer on 17 November 2011 (**a**: left eye, **b**: right eye) and on 21 October 2013 (Day 1, **c**: left eye, **d**: right eye). Deterioration of the visual field defect was detected before prescription of kallidinogenase tablets.

**Figure 3:**
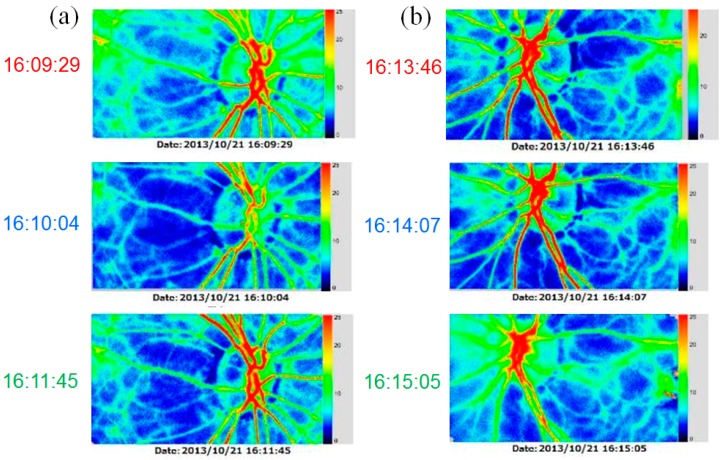
Findings obtained using laser speckle flowgraphy on 21 October 2013 (Day 1, approximately one year before starting prescription of kallidinogenase tablets, **a**: right eye, **b**: left eye).

**Figure 4:**
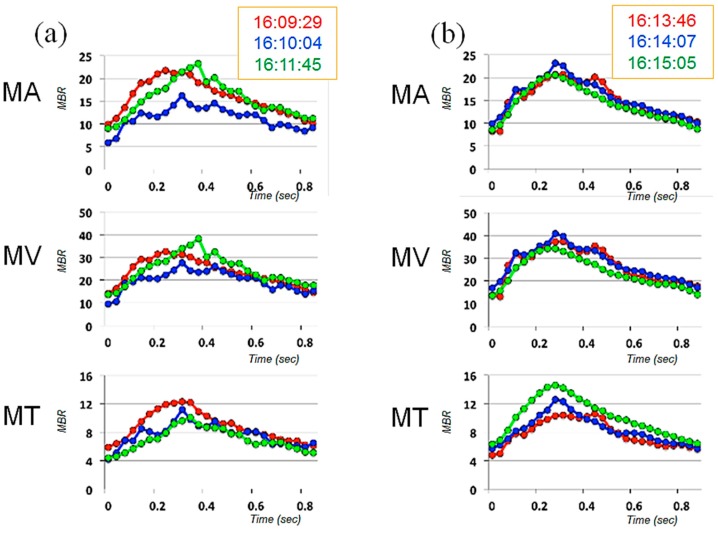
Averaged waveforms of 3 successive measurements for each index of ONH blood flow, obtained on 21 October 2013 (Day 1, approximately one year before starting prescription of kallidinogenase tablets, **a**: right eye, **b**: left eye).

**Figure 5:**
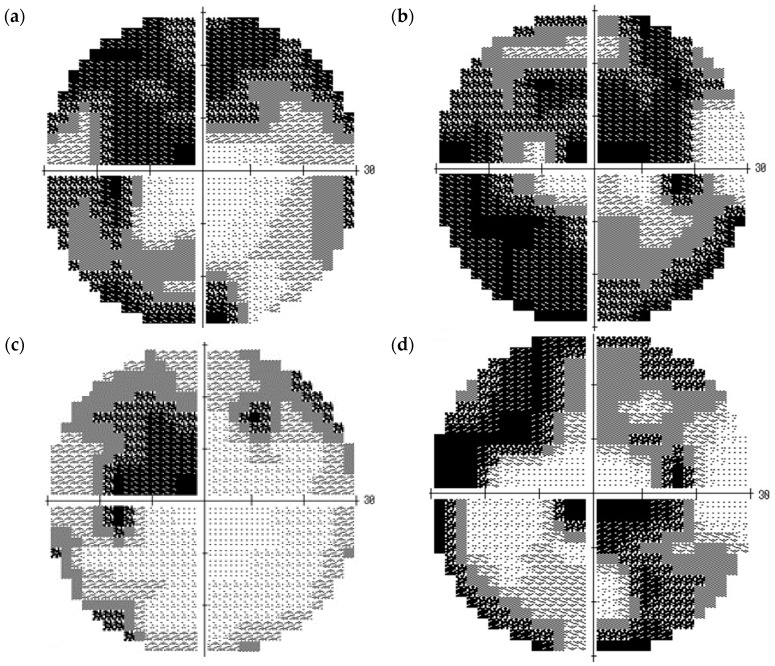

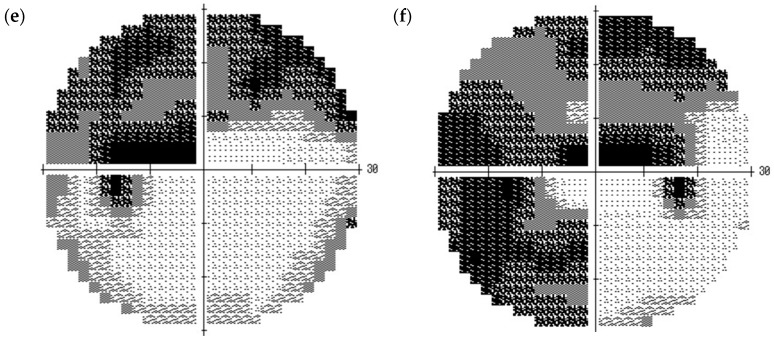
Visual field findings obtained using the Humphrey field analyzer on 19 May 2014 (Day 2, approximately 4 months before prescription of kallidinogenase tablets, **a**: left eye, **b**: right eye), 26 March 2015 (approximately 6 months after starting prescription of kallidinogenase tablets, **c**: left eye, **d**: right eye), and 27 April 2016 (Day 4, approximately 7 months after the patients stopped taking kallidinogenase tablets, **e**: left eye, **f**: right eye).

When she visited our clinic on 21 October 2013 (Day 1, approximately 3 years after the beginning of tafluprost application), her best-corrected visual acuity was 18/20 in the right eye (OD) and 14/20 in the left eye (OS); her IOP was 11 mmHg OD and 13 mmHg OS; and the cup-to disc ratio was 0.8 OD and 0.6 OS (Figure 1). Deterioration of the visual field defect was detected in both eyes when compared with previous results (Figure 2). Measurement of ONH blood flow on LSFG-NAVI (Softcare Co., Ltd., Fukuoka, Japan) indicated an increased fluctuation with a noticeably larger coefficient of variation (CV) than healthy subjects (Figure 3 and Figure 4, Table 1). Approximately 7 months later (Day 2), in addition to the increased fluctuation, her visual field defect deteriorated further (Table 1, Figure 5).

**Figure 6:**
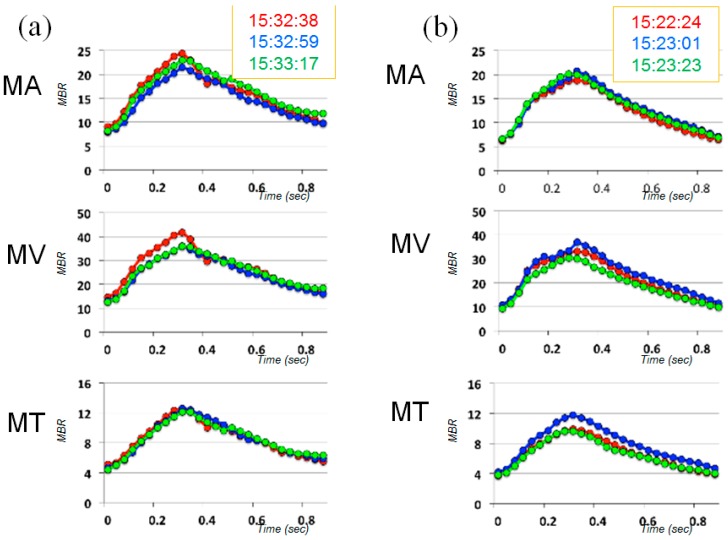
Averaged waveforms of 3 successive measurements for each index of ONH blood flow, obtained on 4 December 2014 (Day 3, approximately 3 months after starting prescription of kallidinogenase tablets, **a**: right eye, **b**: left eye).

**Figure 7:**
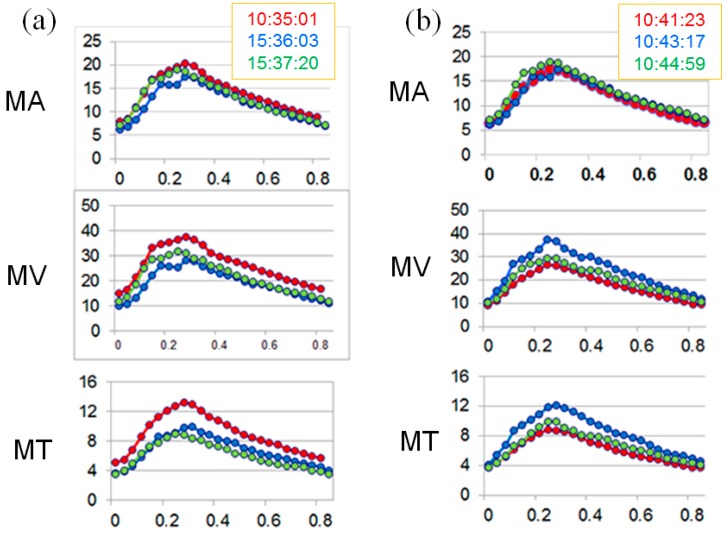
Averaged waveforms of 3 successive measurements for each index of ONH blood flow, obtained on 27 April 2016 (Day 4, approximately 7 months after the patient stopped taking kallidinogenase tablets, **a**: right eye, **b**: left eye).

Kallidinogenase tablets (Sanwa Kagaku Kenkyusho Co., Ltd., Nagoya, Japan) were prescribed for her starting in September 2014. Approximately 3 months later (Day 3), the fluctuation of ONH blood flow was noticeably reduced in both eyes (Figure 6, Table 1). In addition, 6 months after starting prescription of kallidinogenase tablets, her visual field defect was ameliorated in both eyes (Figure 5). However, in September 2015 (one year after starting prescription of kallidinogenase tablets), she stopped taking the kallidinogenase tablets because of discomfort. Seven months later (Day 4), the fluctuation of ONH blood flow increased again (Figure 7, Table 1), and the visual field defect deteriorated (Figure 5).

## 3. Discussion

To the best of our knowledge, this is the first report indicating increased short-term fluctuation of ONH blood flow in a case of NTG, detected by the use of LSFG. This case exhibited progressive visual field defect in spite of stable IOP in the low teens.

We measured ONH blood flow using LSFG-NAVI, an updated model of LSFG. The principle and method for determining ONH blood flow with LSFG-NAVI have been described elsewhere [17,18]. Mean blur rate (MBR) was used as an indicator of blood flow. We obtained the mean MBRs throughout the ONH (MA), the mean MBRs of the ONH vessels (MV), and the mean MBRs of the ONH tissue (MT). The short-term fluctuation of ONH blood flow was evaluated based on the CV calculated from 3 continuous measurements of MA, MV, and MT. At the first measurement, the CV in both eyes was noticeably larger than in healthy controls [16], demonstrating increased fluctuation of ONH blood flow. These large CVs were reproduced later; although the CVs were critically reduced and the visual field defect was ameliorated after the patient was administered kallidinogenase tablets, after the medication was discontinued, the CVs increased again and the visual field defect deteriorated. Based on the clinical changes over time, the increased fluctuation of ONH blood flow seemed to be associated with the development of glaucomatous visual field defect in this case. However, because this is a report of only one case, further investigation of multiple cases is necessary in the future. Though we did not acquire any data regarding a short-term fluctuation of systemic blood pressure at the time of ONH blood flow measurement, it is possible that the patient’s systemic vascular dysregulation is attributed to the fluctuation of ONH blood flow.

## Figures and Tables

**Table 1:** Coefficients of variation for indices of optic nerve head (ONH) blood flow.

Time	MA		MV		MT	
	Right	Left	Right	Left	Right	Left
Day 1	15.3	5.4	10.9	9.6	10.5	11.2
Day 2	8.4	10.1	8.9	10.0	19.3	16.5
Day 3	5.8	3.8	4.9	7.8	2.9	8.2
Day 4	8.7	6.7	10.7	14.6	21.0	14.0
Normal [16] (Mean ± SD)	2.9 ± 2.1		1.9 ± 1.2		2.1 ± 1.1	

MA: the mean MBRs throughout the ONH, MV: the mean MBRs of the ONH vessels, MT: the mean MBRs of the ONH tissue. Day 1: 21 October 2013; Day 2: 19 May 2014; Day 3: 4 December 2014; Day 4: 27 April 2016.
